# Synthesis of Gemcitabine-Loaded PLGA Microparticles with Green Solvents

**DOI:** 10.1021/acsomega.5c06385

**Published:** 2025-07-24

**Authors:** Irene Alvarez, Cristina Gutiérrez, Ignacio Gracia, Juan Francisco Rodríguez, María Teresa García

**Affiliations:** † Department of Chemical Engineering, 16733 University of Castilla-La Mancha, Facultad de C.C. Químicas, Avda. Camilo José Cela 12, 13071 Ciudad Real, Spain; ‡ Department Hydrogen and Power to X, Iberian Centre for Research in Energy Storage (CIIAE), Avda. Universidades s/n, 10003 Caceres, Spain

## Abstract

The use of polymeric microparticles is widely recognized as an effective strategy for enhancing the bioavailability and biodistribution of both lipophilic and hydrophilic medications. In this study, PLGA microparticles loaded with the anticancer drug gemcitabine were synthesized by using a double emulsion process known as water-in-oil-in-water solvent evaporation. Notably, this is the first time that ethyl lactate, an FDA-approved green solvent, has been used for the microparticle synthesis. For comparison, other solvents, such as ethyl acetate and dichloromethane, were also tested. The smallest particle size was achieved, regardless of the PLA:PGA ratio of the polymer, when using ethyl lactate under the following operational conditions: a high homogenizer speed (12,000 rpm) and an initial polymeric solution concentration of 1.5% w/v. Under these conditions, the encapsulation efficiency reached 45% for PLGA 50:50 and 35% for PLGA 75:25. Microparticle analysis revealed a homogeneous distribution (100–150 μm range) and a spherical shape. Furthermore, Fourier transform infrared (FTIR) spectroscopy confirmed the presence of gemcitabine in the microparticles.

## Graphical abstract

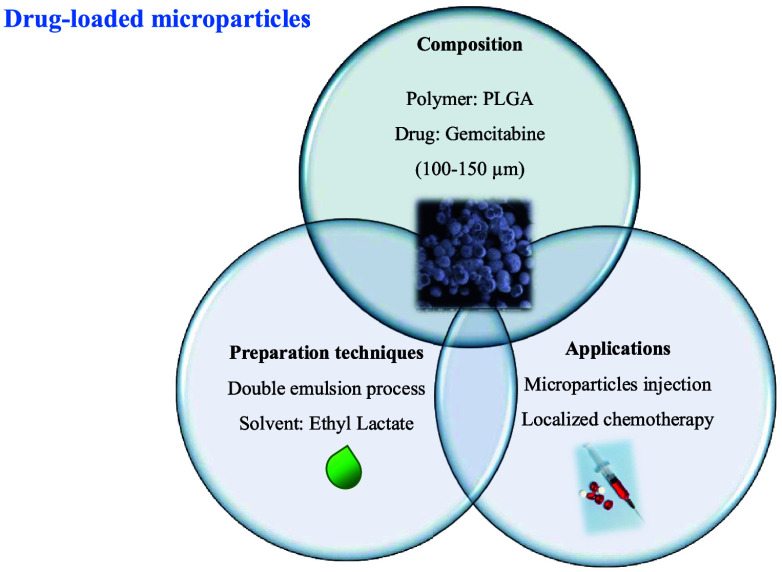

## 1 Introduction

Gemcitabine (2′,2′-difluorodeoxycytidine) (GEM) is a widely used anticancer drug, primarily employed in the treatment of solid tumors.^1,2^ It is commonly administered as an intravenous infusion against several human malignant tumors, including pancreatic,^3^ ovarian,^4^ lung,^5^ breast,^6^ and bladder.^7^ Its half-life in systemic circulation is relatively short, ranging from 8 to 17 min, necessitating high, frequent doses, which can lead to myelosuppression and hepato- and nephrotoxicity.^8^ Therefore, new therapeutic approaches are essential to improve the efficacy of treatment while minimizing these adverse effects.

One promising approach is the use of controlled release systems, which deliver the drug in a predesigned form directly to the target tissue. This method can inhibit tumor growth while reducing the toxic effects typically associated with chemotherapy.^9^

A particularly interesting treatment being explored to reduce side effects is the direct injection of drug-loaded microparticles. These injections can deliver cancer drugs directly to the surrounding tissue following surgical resection^10^ providing localized chemotherapy. This approach allows for controlled and sustained release of the drug, which can maintain therapeutic levels in the targeted area, reducing systemic toxicity^11^ and hypersensitivity reactions.^12^

Controlled release systems, whether microparticles or scaffolds obtained from biodegradable polymers such as poly­(d,l-lactic acid) (PLA) or poly­(d,l-lactide-*co*-glycolide) (PLGA), are widely researched for cancer treatment.^13–24^ These polymers, both PLA and PLGA, have been approved by the FDA for use in pharmaceutical applications as their degradation products are CO_2_ and H_2_O, which are eliminated from the human organism by metabolic pathways.^25^ Among these polymers, PLGA is more widely used due to its enhanced compatibility with the requirements of patients.^26–28^

There are several methods for synthesizing microparticles that are suitable for developing drug delivery systems from PLGA polymer, such as solvent evaporation,^29,30^ coacervation,^31,32^ or spray drying.^33,34^ In these methods, selecting an appropriate solvent to dissolve the polymer is crucial, as its characteristics—such as boiling point and solvency power—impact both the process and the final properties of the microparticles.

From a scientific perspective, an appropriate solvent should be capable of dissolving hydrophobic PLGA polymers, be water-insoluble to facilitate emulsion formation, and be volatile for easy removal during or after manufacturing. In the case of GEM-loaded microparticles, most studies use toxic organic solvents, such as dichloromethane, chloroform, or acetone in the synthesis process.^13,35–38^ Dichloromethane is listed on the ATSDR (Agency for Toxic Substances and Disease Registry) 2019 Substance Priority List, ranking 90th out of 275 hazardous compounds. Additionally, the US Department of Health and Human Services (DHHS) classifies methylene chloride as a human carcinogen. The European Medicines Agency (EMA) has established a concentration limit of 600 ppm for DCM in pharmaceutical products. Chloroform^39–41^ has also been widely used; however, due to its significant hazards, its use has been steadily declining. The EMA recommends a concentration limit of 60 ppm for chloroform in pharmaceutical products. Acetone poses a lower risk to human health, at levels typically accepted in pharmaceuticals. Although long-term toxicity studies are lacking, short-term data indicate that it is less toxic. The EMA considers residual acetone levels up to 5000 ppm acceptable.^42^

Due to the toxicity and environmental impact of traditional solvents, researchers have increasingly explored safer, more sustainable alternatives for the development of pharmaceutical microparticles. In this study, nonloaded PLGA microparticles were synthesized using oil-in-water solvent evaporation, and GEM-loaded PLGA microparticles were synthesized via the double emulsion process (water-in-oil-in-water solvent evaporation), utilizing ethyl lactate (EL) as the solvent for the first time. EL, a biobased, fully degradable green solvent, has gained significant attention as an eco-friendly and economically viable alternative to traditional solvents. It has also been compared to other solvents such as ethyl acetate (EA) and dichloromethane (DCM). To assess the effectiveness of these solvents, we analyzed the size and shape of the resulting microparticles. Additionally, the impact of variables such as the PLA:PGA ratio, the initial polymer concentration, agitation speed, and homogenizer settings on the microparticle size was examined. Notably, the use of EL in this context is a novel contribution, as no prior studies in the literature have utilized this solvent in the synthesis of GEM-loaded PLGA microparticles. This work aims to develop a novel and environmentally friendly method for the synthesis of PLGA microparticles from an engineering perspective. Further pharmacokinetic and toxicological studies of the synthesized particles are necessary in future investigations.

## 2 Experimental Section

### 2.1 Materials

Poly­(lactic-*co*-glycolic) acid (PLGA) with different lactide/glycolide (average molecular weight 17,000 g/mol) ratios was used for the synthesis of the microparticles. PLGA5050 (50 mol % lactic acid, 50 mol % glycolic acid) and PLGA7525 (75 mol % lactic acid, 25 mol % glycolic acid) were supplied by Corbion Purac and used as received. Poly­(vinyl alcohol) (PVA) of molecular weight 61,000 g/mol was purchased from Sigma-Aldrich (Spain). Ethyl acetate, ethyl lactate, and dichloromethane were also acquired from Sigma-Aldrich (Spain) and used as received. GEM hydrochloride was supplied by Sigma-Aldrich (Spain).

### 2.2 Synthesis of Microparticles

In this work, two methods for synthesizing drug-free or drug-loaded microparticles were developed: oil-in-water solvent evaporation and water-in-oil-in-water solvent evaporation. The hydrophilicity of GEM is a requirement for the application of the double emulsion method.

#### 2.2.1 Oil-in-Water Solvent Evaporation

Polymeric nonloaded microparticles were prepared by the oil-in-water (o/w) emulsion solvent evaporation technique. The corresponding amount of polymer was dissolved in 10 mL of solvent to prepare the polymeric solutions. The solution was added to 100 mL of the 1% (w/v) PVA aqueous solution by stirring at 600 or 1200 rpm to volatilize the organic solvent. After 24 h of stirring at ambient temperature, the aqueous suspension was centrifuged at 3000 rpm for 15 min. Finally, the microparticles obtained were washed three times with distilled water, filtered, and stored at −10 °C.

#### 2.2.2 Water-in-Oil-in-Water Solvent Evaporation

GEM encapsulated microparticles were obtained by the double emulsion method because this compound is a hydrophilic drug that can be dissolved in water but not in organic solvents. This process is also known as water-in-oil-in-water (w/o/w) solvent evaporation. First, 10 mg of the drug was dissolved in 0.2 mL of water, and this solution was added to the polymer solution prepared as in the o/w method. Then, this mixture was homogenized (CAT Undrive X 1000 D) at 5600 or 12,000 rpm for 30 s. This emulsion was poured dropwise into 100 mL of the 1% (w/v) PVA aqueous solution and was homogenized for 30 s. This solution was then stirred at 600 or 1200 rpm to volatilize the organic solvent. To obtain microparticles free of traces of solvent and to eliminate the nonencapsulated GEM, microparticles were centrifuged, washed three times with distilled water, filtered, and stored at −10 °C.

### 2.3 Characterization of Microparticles

#### 2.3.1 Microparticles Size and Morphology

The size and morphology of the microparticles were studied by scanning electron microscopy (SEM) using Quanta 250 equipment with a wolfram filament operating at a working potential of 10 kV (FEI Company). Motic Images 2.0 software was used to analyze the mean particle diameter, and homogeneity was calculated from the standard deviation of the sample based on the SEM images.

#### 2.3.2 Thermal Analysis

Differential scanning calorimeter (DSC Q1000 TA Instruments) scans were made using an initial heating at 10 °C/min up to 100 °C to release thermal and absorption history and to provide a better fit in the crucible. The samples were then annealed for 10 min, cooled at the same rate down to 0 °C by using a stream of liquid nitrogen, and annealed for another 10 min. *T*_g_ measurements were carried out during the second heating, and it is identified from the change in heat flow resulting from a change in heat capacity at the transition temperature during each scan.

The residual amount of solvent present in the microparticles was determined by thermogravimetric analysis (TA-DSC Q 600). All analyses were carried out in a nitrogen atmosphere with a flow rate of 100 mL/min. Weight loss due to solvent volatilization (∼[100–170] °C) and polymer degradation (∼350 °C) was recorded in the thermograph as a function of temperature. The presence of GEM was further confirmed by its degradation (∼280 °C). The samples (3–10 mg) were heated from room temperature to 450 °C at a heating rate of 10 °C/min. The data were analyzed with the universal analysis software TA 2000.

#### 2.3.3 FTIR

The Fourier transform infrared (FTIR) spectra of PLGA polymer, nonloaded PLGA microparticles, GEM, and drug-loaded PLGA microparticles were recorded on a JASCO FT/IR 4600. It was used for chemical analyses of the functional groups present in the microparticles. IR spectra of samples were obtained in the range from 4000 to 400 cm^–1^, with a resolution of 4.0 cm^–1^ and 64 scans.

#### 2.3.4 Encapsulation Efficiency

The method for estimating the encapsulation efficiency consisted of dissolving the polymeric matrix in acetonitrile and then solubilizing the amount of drug entrapped into the microparticles in phosphate-buffered saline (PBS).^13^ The amount of GEM in PLGA was estimated by adding 10 mg of microparticles in 1 mL of acetonitrile and shaking it vigorously to solubilize the polymer to extract out the drug, followed by addition of 10 mL of PBS to solubilize the drug. The amount of GEM was measured by a ultraviolet (UV) spectrophotometer (HELIOS ZETA UV–vis) at a wavelength of 268 nm. The yield of the encapsulated drug was measured as

1 $$\%\;encapsulation\;efficiency=\frac{amount\;of\;drug\;encapsulated}{amount\;of\;drug\;added}\times 100$$

## 3 Results

The outcomes of this work have been separated into two sections: the creation of unloaded microparticles by o/w solvent evaporation and the synthesis of GEM-loaded microparticles by the w/o/w technique.

### 3.1 Formation of Nonloaded Drug Microparticles by o/w Method

To replace conventional organic solvents with a less toxic alternative, this study synthesized microparticles for parenteral drug delivery using green solvents such as EA and EL, as well as the traditional solvent DCM.

For controlled drug release applications, the size and size distribution of microparticles are critical factors, as these parameters can influence encapsulation efficiency (EE), product injectability, *in vivo* biodistribution, and the release kinetics of the encapsulated drug.^43,44^ Additionally, these characteristics impact therapeutic efficacy and the profile of adverse effects.^45^ Although the term microparticle is used to describe spherical particles between 1 and 1000 μm in diameter, in the design of drug delivery systems based on microparticles, optimal drug release profiles are typically achieved using microparticles with diameters ranging from 10 to 200 μm.^46^ Microparticles with diameters smaller than 10 μm are at a higher risk of being phagocytosed by immune cells,^47^ while those exceeding 200 μm may trigger an immune response and inflammation.^47^

This study investigated the influence of four variables and their levels on microparticle size and their distribution (Table 1) using a full factorial design of experiments (DOE) with a total of 24 experiments. All experiments followed the same procedure and were conducted in a random order to minimize the effects of hidden variables.

**Table 1:** Production of Nonloaded Drug Microparticles

independent variables	experimental range	levels	dependent variables	*p*-value
solvent	EL-EA-DCM	(−1)–(0)–(1)	particle size	0.0001
polymer concentration organic solution (% w/w)	0.5–1.5	(−1)–(1)		0.7914
agitation speed (rpm)	600–1200	(−1)–(1)		0.1879
ratio PLA:PGA	50:50–75:25	(−1)–(1)		0.8340

Table 2 shows the obtained results. The average particle size and its standard deviation were determined by analyzing multiple microphotographs in each experiment. It is important to note that the magnifications of these three photographs were adjusted according to particle size to obtain better resolution and visualization of the microparticles.

**Table 2:** Experimental Conditions for Each Run, Size of Particles, and Standard Deviation

run	solvent	conc. (% w/v)	agitation (rpm)	ratio PLA:PGA	particle aggregation	particle size (μm)	std. dev. (μm)
1	EL	0.50	600	50:50	yes	20.02	2.17
2	EL	1.50	600	50:50	yes	59.54	8.17
3	EL	0.50	1200	50:50	no formation		
4	EL	1.50	1200	50:50	yes	5.08	1.18
5	EL	0.50	600	75:25	yes	3.98	2.72
6	EL	1.50	600	75:25	yes	4.46	1.73
7	EL	0.50	1200	75:25	no formation		
8	EL	1.50	1200	75:25	no formation		
9	DCM	0.50	600	50:50	yes	13.68	4.58
10	DCM	1.50	600	50:50	no	44.17	11.81
11	DCM	0.50	1200	50:50	yes	11.80	3.53
12	DCM	1.50	1200	50:50	no	29.30	13.11
13	DCM	0.50	600	75:25	no	60.48	8.47
14	DCM	1.50	600	75:25	no	42.54	12.27
15	DCM	0.50	1200	75:25	yes	12.11	5.08
16	DCM	1.50	1200	75:25	yes	26.02	6.47
17	EA	0.50	600	50:50	no	1199.74	271.23
18	EA	1.50	600	50:50	no	1320.14	266.74
19	EA	0.50	1200	50:50	no	1076.25	77.48
20	EA	1.50	1200	50:50	no	829.46	79.25
21	EA	0.50	600	75:25	no	1904.71	333.29
22	EA	1.50	600	75:25	no	1417.00	415.86
23	EA	0.50	1200	75:25	no	756.63	138.90
24	EA	1.50	1200	75:25	no	772.73	60.45

In the analysis of variance (ANOVA), a factor is considered statistically significant when its *p*-value is below 0.05. Table 1 presents the *p*-values for each evaluated factor, and Figure 1 illustrates the estimated effects of the parameters on the response variables (particle size and size distribution) in descending order of importance. The length of each bar is proportional to the standardized effect, and the vertical line indicates the threshold for statistical significance at a 95% confidence level.

**Figure 1:**
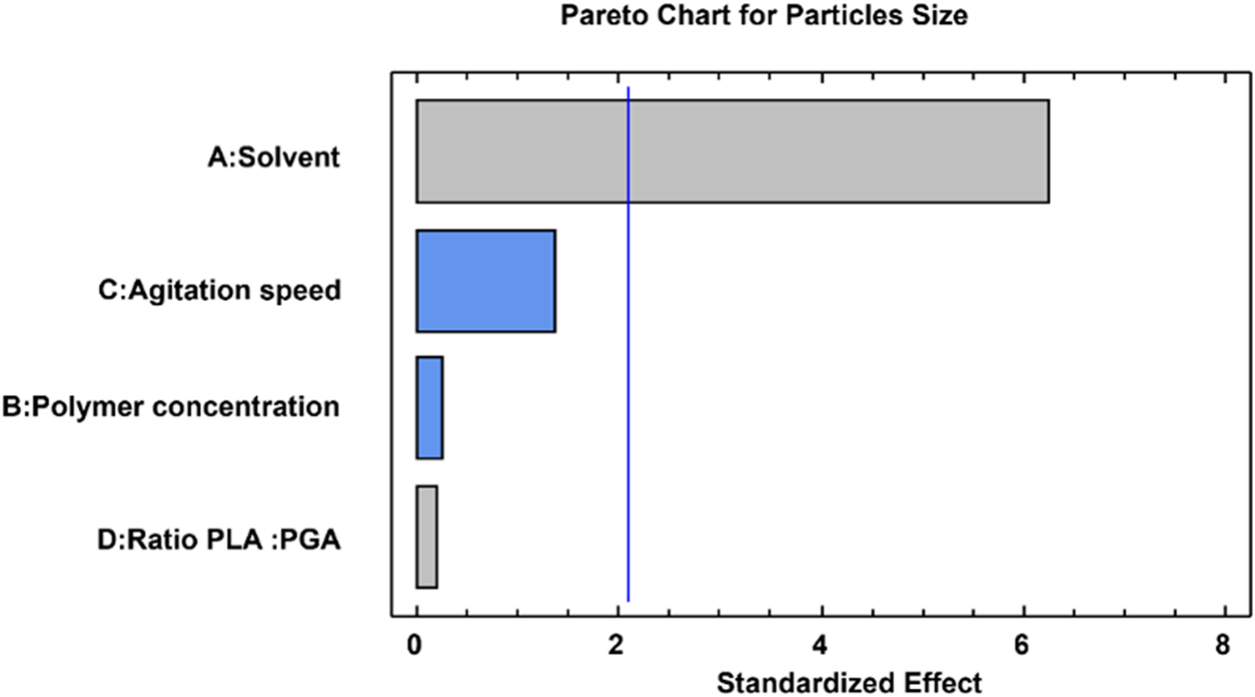
Pareto chart for the standardized effects of the independent variables (A) solvent, (B) initial polymer concentration, (C) agitation speed, and (D) Ratio PLA: PGA on the mean particle size (blue bars: favorable effect on the response; gray bars: non-favorable effect on the response).

First, it can be observed that the solvent was the most significant factor controlling the particle size, as depicted by the length of the bars (Figure 1) and their *p*-values being less than the a priori value of 0.05. Other factors, such as agitation speed, polymer concentration in organic solvents, and the PLA:PGA ratio in the polymer, also had an effect, but their effects were not statistically significant (*p* > 0.05).

Comparing the solvents, it can be observed that EL produces smaller particles than those obtained with DCM, but both solvents produce particles within the size range indicated for drug administration via the parenteral route (10–200 μm).^48^ However, most particles obtained with EA are in millimeters. This is probably because, in the particle synthesis with EA, higher agitation speeds are needed to break the emulsion and form microdroplets.^49^

On the other hand, when EL is used, the particles exhibit an irregular shape or, in some cases, aggregation occurs, suggesting the occurrence of coalescence phenomena during polymer precipitation and particle formation.^50^ This is because, when the dispersed phase (PLGA in EL) is introduced directly into the continuous aqueous phase, the sudden extraction of a large amount of organic solvent from the dispersed phase leads to the precipitation of the polymer as an aggregate.^51^ This fact is because EL has a higher solubility in water than DCM and EA, which are only partially miscible.

Lastly, the particles synthesized with DCM exhibit a smoother surface. The difference in morphology (rough or smooth surface) could be attributed to the solvent evaporation rate. DCM evaporates faster than EA or EL because it has a lower boiling point (39.6 °C versus 77.1 °C for EA and 154 °C for EL), making its evaporation more rapid and efficient.^49^

With respect to the agitation speed, it also affected the average particle size, but its effects were not statistically significant (*p* > 0.05). As can be observed, the experiments conducted show that an increase in this variable from 600 to 1200 rpm decreases both the average particle size and its heterogeneity. This can be attributed to the increased turbulent kinetic energy involved in the droplet fragmentation process. At lower agitation speeds, the primary colloidal droplets tend to coalesce, resulting in the formation of larger coacervated particles and causing macroscopic phase separation.^52,53^

Furthermore, as depicted in the Pareto chart (Figure 1), no clear difference in microparticle size and formation is observed with respect to the influence of the initial polymer concentration in the organic phase and the PLA-to-PGA ratio. However, the latter parameter may play a crucial role in controlling the degradation rate, as it is known that a higher glycolide content results in faster degradation.^25^

Figure 2 shows SEM images of experiments 4, 12, and 20. These experiments demonstrate that under identical experimental conditions (initial polymer concentration of 1.50% w/v, stirring speed of 1200 rpm, and using PLGA5050 as the polymer), the smallest microparticles are obtained with EL, followed by those produced with DCM. However, the microparticles generated using DCM exhibit the highest uniformity. Conversely, the particles obtained with EA exhibit a size that is excessively large, rendering them unsuitable for application in controlled release systems.

**Figure 2:**
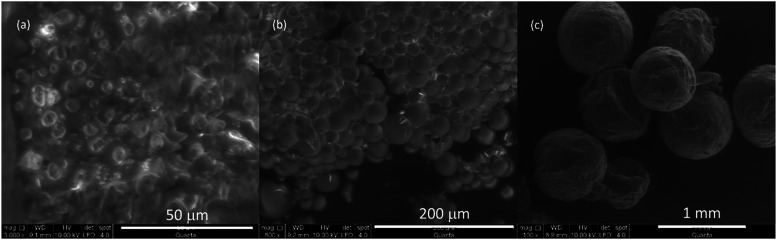
SEM images: (a) Run 4 (operation conditions: EL as solvent, 1.50% w/v 1200 rpm, PLGA5050), magnification ×3000, (b) Run 12 (operation conditions: DCM as solvent, 1.50% w/v 1200 rpm, PLGA5050), magnification ×800, (c) Run 20 (operation conditions: EA as solvent, 1.50% w/v 1200 rpm, PLGA5050), magnification ×100.

To continue the study, DCM was discarded despite the production of particles with a suitable and homogeneous size distribution due to its nature as a harmful organic solvent. In contrast, EA and EL were selected to produce drug-loaded particles due to their benefits as green solvents, introducing an additional stirring step to further reduce the droplet size generated during the emulsion process, prevent the formation of aggregates, and improve the formation of subvisible particulates.

### 3.2 Encapsulation of GEM by w/o/w Method

#### 3.2.1 Size and Morphology of Microparticles

As previously mentioned, size is a key factor in microparticle synthesis, as a specific size is required for parenteral drug delivery.

The effects of five parameters and their levels on microparticle size were investigated using a full factorial design (Table 3) with a total of 32 experiments. The five factors were: solvent, polymer concentration in the organic solution, first agitation step, second agitation step, and PLG:PGA ratio. To minimize the influence of hidden variables, all tests were conducted following the same experimental protocol and in a random order.

**Table 3:** Production of GEM-Loaded Microparticles

independent variables	experimental Range	levels	dependent variables	*p*-value
solvent	EL-EA	(−1)–(1)	particles size	0.0000
polymer concentration organic solution (% w/v)	0.5–1.5	(−1)–(1)		0.1803
1st step of agitation (rpm)	5600–12000	(−1)–(1)		0.0463
2° step of agitation (rpm)	600–1200	(−1)–(1)		0.9239
ratio PLA:PGA	50:50–75:25	(−1)–(1)		0.8340

Table 3 also presents the *p*-values, while Figure 3 shows the standardized Pareto analysis. Table 4 presents the microparticle size and standard deviation for each experimental run along with SEM microphotographs. The average particle size and its standard deviation were calculated based on the analysis of multiple microphotographs from each experiment. Furthermore, for each experiment in which microparticles were obtained, an encapsulation efficiency study was performed.

**Table 4:**
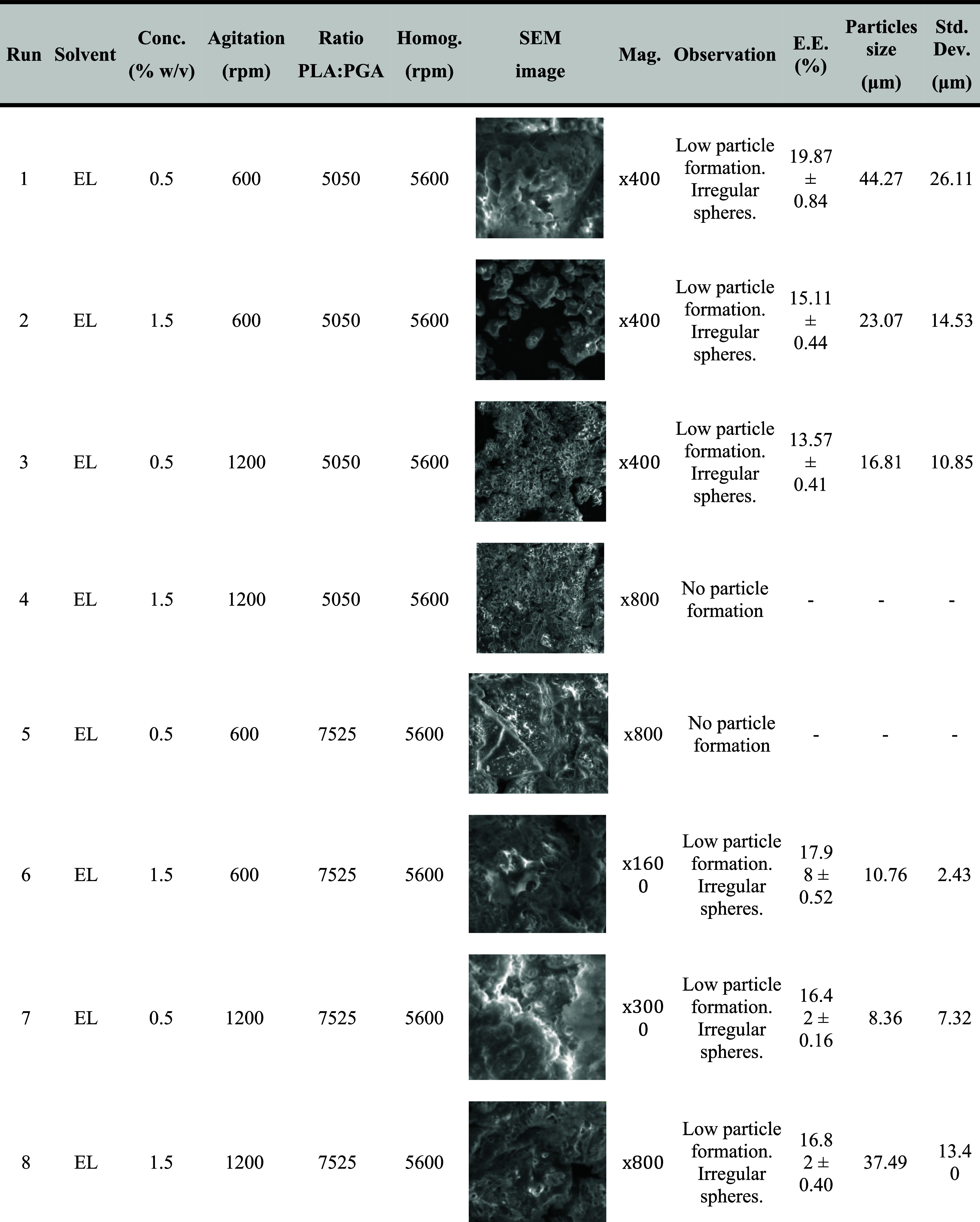

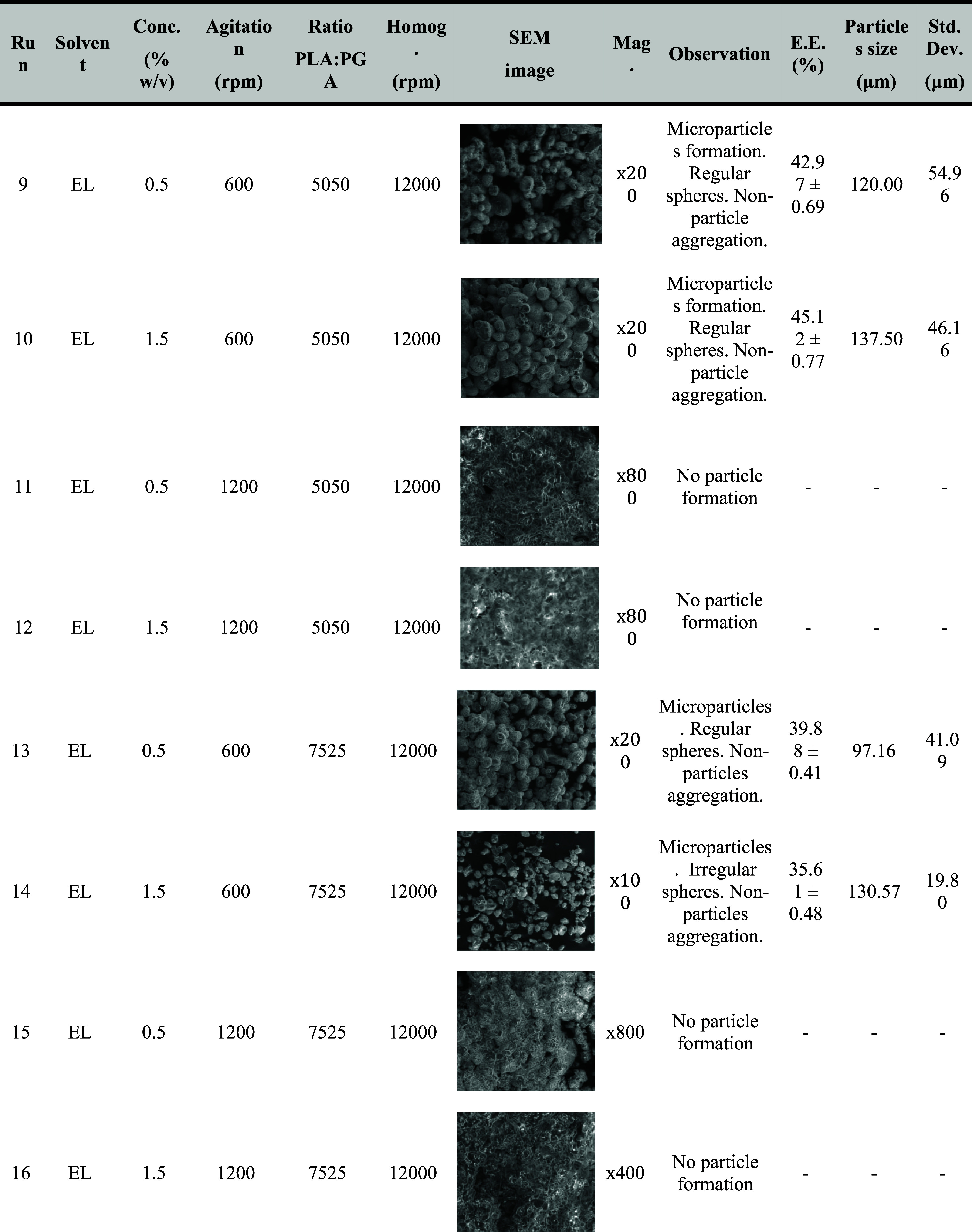

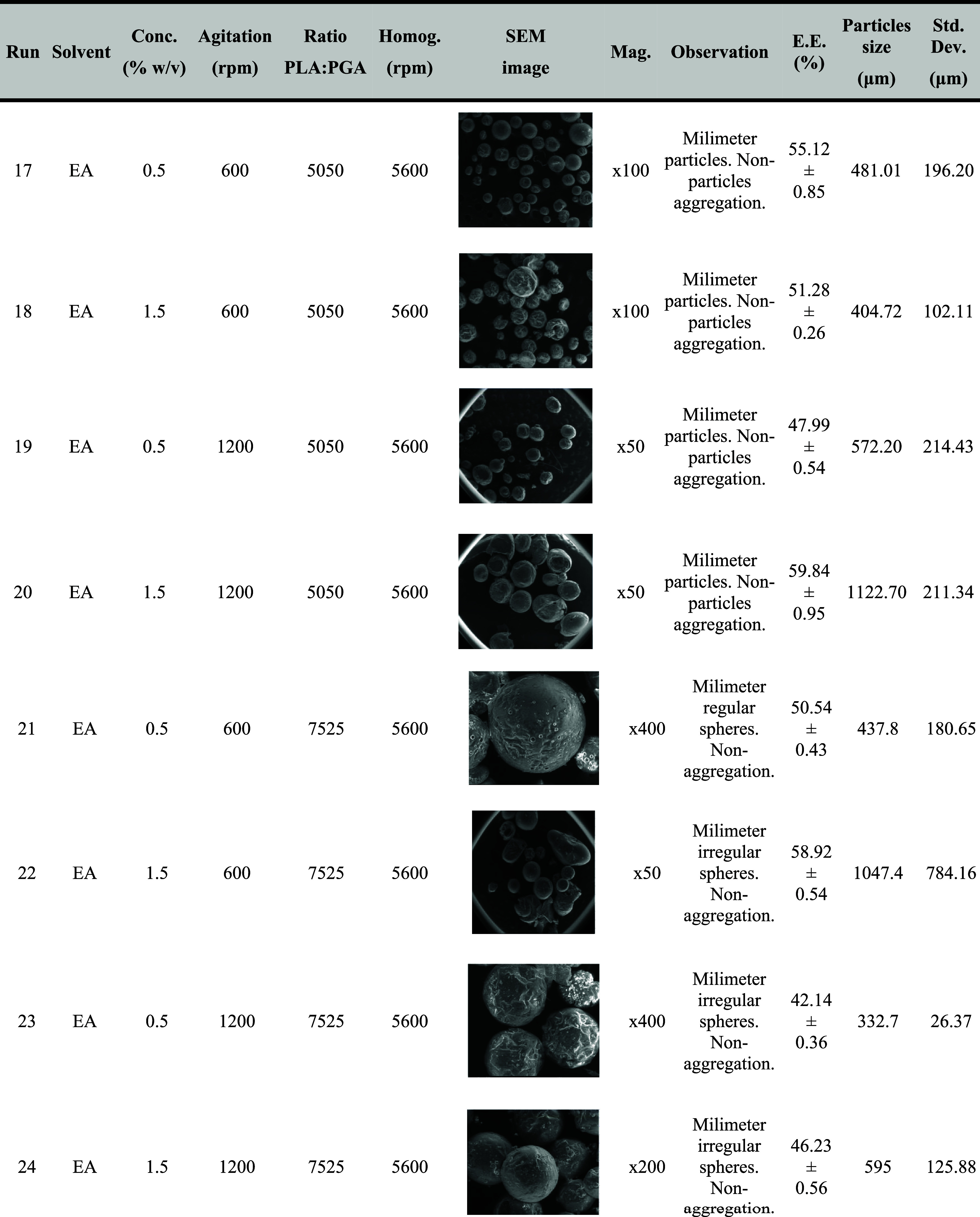

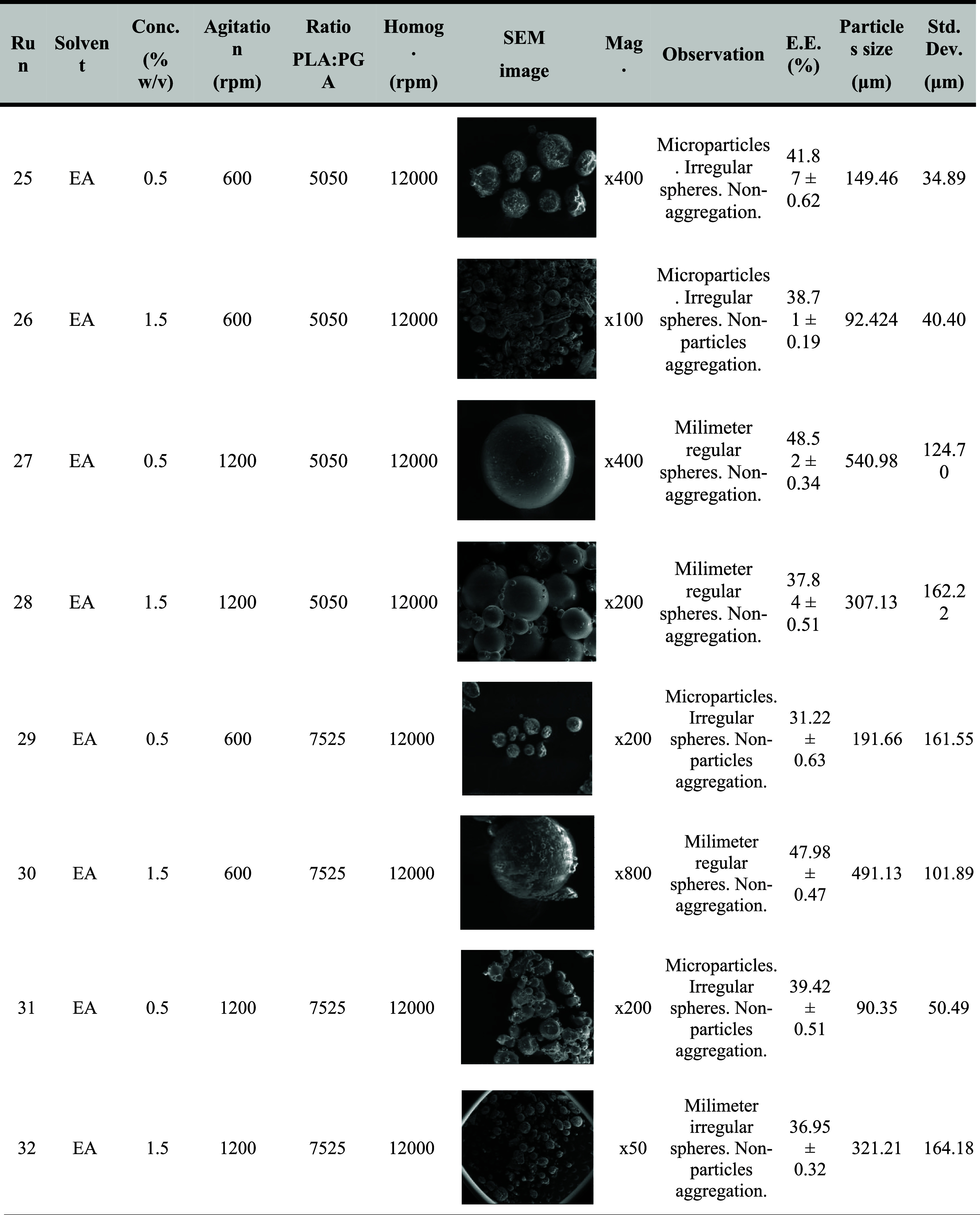
Experimental Conditions for Each Run, SEM Images and Their Magnification, Size of Microparticles Formed, Its Deviation, and GEM Encapsulation Efficiency

**Figure 3:**
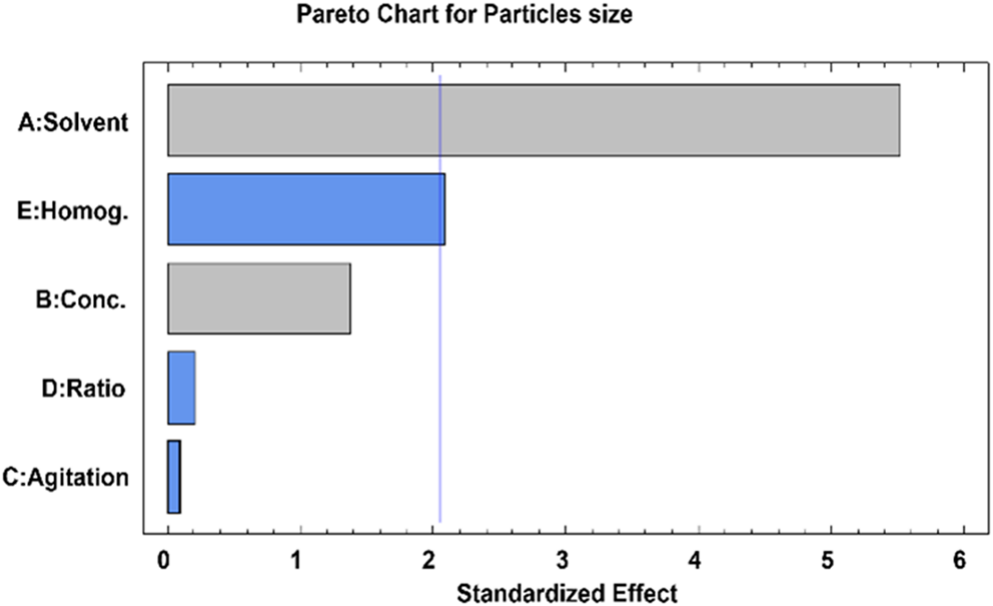
Pareto chart for the standardized effects of independent variables: (A) solvent, (B) initial polymer concentration, (C) second agitation step and (D) ratio PLA:PGA, (E) first agitation step: homogenizer on the mean particle size (blue bars: favorable effect on the response; gray bars: non-favorable effect on the response).

From Table 3 and Figure 3, it was observed that particle size was affected only by the factors with *p*-values below the significance threshold (*p* < 0.05), which, in decreasing order, were the solvent used and the first agitation step. All other parameters were not significant at a 95% confidence level.

When EA is used as a solvent, GEM-loaded particles are produced within a size range of 100 to 1000 μm. Although this is smaller than the unloaded particle size, it still does not fall within the optimal size range for drug administration via the parenteral route (10–200 μm^48^). In contrast, the use of an EL results in a greater number of GEM-loaded microparticles of the appropriate size and a homogeneous size distribution.

Regarding the next significant factor, a generally inverse relationship between the agitation rate and mean particle size was observed. As the agitation rate increased from 5600 to 12,000 rpm, the particle size of the microparticles decreased, particularly in experiments where EA was used as the solvent. It is widely recognized that the degree of agitation influences the stability of the emulsion. Proper agitation provides the necessary energy to the system, facilitating the breakdown of the droplets in the dispersed phase, resulting in smaller particles.^54^

With respect to the increase in polymer concentration in the organic phase, it does not significantly impact particle size; however, as the concentration increases, a slight increase in microparticle size is observed. Polymer concentration influences the internal phase viscosity, meaning that a higher polymer concentration results in an increased viscosity of the internal phase. Consequently, during the emulsion formation step, the internal phase breaks into larger droplets.^55^ Moreover, this extra stirring step also promotes solvent evaporation, leading to faster elimination and reducing the time required to form many spherical microparticles with a smooth surface.

The surface morphology of GEM-loaded PLGA microparticles revealed a clear influence of both the solvent and processing conditions on the particle roughness. Microparticles produced with ethyl acetate (EA) consistently exhibited higher surface roughness compared with those prepared with ethyl lactate (EL), likely due to faster solvent evaporation and phase separation dynamics. Moreover, increasing the agitation speed from 600 to 1200 rpm led to more irregular and rougher surfaces regardless of the solvent used. In contrast, the application of a second step involving high-speed homogenization at 12,000 rpm significantly improved particle morphology, resulting in more spherical and smoother surfaces. These findings indicate that the combination of EL as solvent and homogenization at 12,000 rpm represents the most favorable condition for producing microparticles with low surface roughness.

Surface roughness plays a critical role in the behavior of the polymeric microparticles. The adsorption of a polymer solution onto a substrate is influenced by mechanical entrapment, which can be enhanced by the presence of nanoporosity or surface irregularities. In the case of drug-loaded microparticles, such as those encapsulating GEM, surface roughness directly impacts the drug release profiles. A low surface roughness is particularly desirable for achieving extended drug release.^56^ Irregularities on the particle surface increase the effective surface area, which in turn accelerates diffusion and promotes the faster release of the encapsulated drug. Therefore, optimizing fabrication parameters to minimize surface roughness is essential to ensure controlled and sustained drug delivery.^57^ Finally, the particle size is unaffected by the PLA-to-PGA ratio and the second agitation step.

#### 3.2.2 Encapsulation Efficiency

First, an FTIR analysis was carried out to verify whether GEM had been properly incorporated into the PLGA microparticles. The FTIR spectra of the PLGA polymer, GEM, and GEM-loaded PLGA microparticles are shown in Figure 4. In the FTIR spectrum of the PLGA polymer, the peak at 1750 cm^–1^ was observed due to the absorbance of the carbonyl group in the PLGA matrix, and the peaks at 2991 and 2952 cm^–1^ correspond to C–H bending vibrations. The FTIR spectrum of GEM displayed bending vibrations of amines at 1700 and 1679 cm^–1^, and the amine stretch at 3256 cm^–1^. The spectrum of GEM-loaded PLGA microparticles showed the carbonyl group peak at 1750 cm^–1^ and C–H bending vibrations at 2998 and 2946 cm^–1^. The peak at 1633 cm^–1^ reinforced the presence of an amide bond. These results are consistent with previous research and confirm the formation of PLGA-GEM conjugates.^1,58–61^

**Figure 4:**
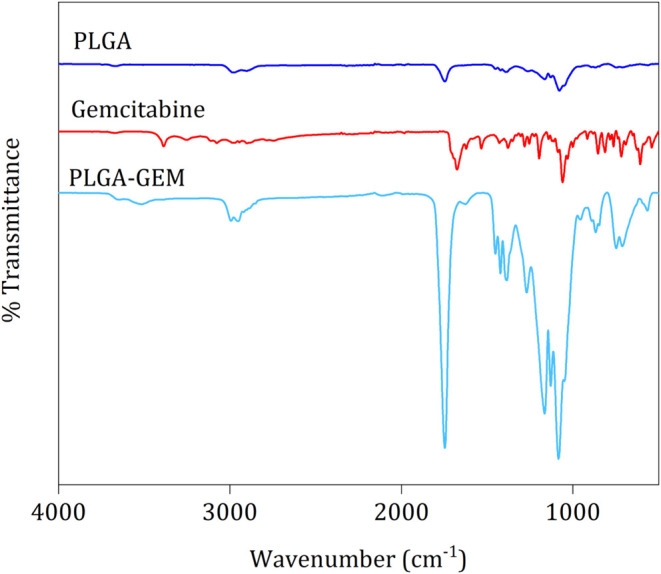
FTIR spectra of PLGA polymer and GEM and PLGA-GEM-loaded microparticles.

Table 4 presents the encapsulation efficiency for the experiments in which a significant formation of microparticles was achieved. It can be observed that when EL is used as the solvent, the encapsulation efficiency ranges from 10 to 45%, whereas with EA, it varies between 30 and 55%. This is because encapsulation efficiency increases with particle size.^62^ Consequently, since larger particles are obtained with EA, a higher amount of gemcitabine is encapsulated. On the other hand, the encapsulation efficiency achieved with EL was greater in the experiments where the microparticles were in the 100–150 μm range^48^ and exhibited higher homogeneity.

Also, it can be observed that the concentration of PLGA in the organic phase affects the encapsulation efficiency of the microspheres. A low concentration of PLGA results in an organic phase with low viscosity, which allows drugs (especially small-molecule drugs) to escape into the aqueous phase, reducing the effectiveness of encapsulation. Conversely, a high PLGA concentration increases the viscosity of the organic phase, hindering the transport of drugs into the aqueous phase while enhancing encapsulation efficiency.^63^

In summary, the smallest particle size was obtained, regardless of the PLA:PGA ratio, using EL under the following conditions: high agitation step (homogenization at 12,000 rpm) and an initial polymeric solution concentration of 1.5% w/v. Under these conditions, encapsulation efficiencies of 45% for PLGA 50:50 and 35% for PLGA 75:25 were achieved. Microparticle studies revealed a homogeneous distribution and a spherical shape. Table 5 shows the results obtained for the particles under optimal conditions, including their glass transition temperature (Tg) and residual solvent content.

**Table 5:**
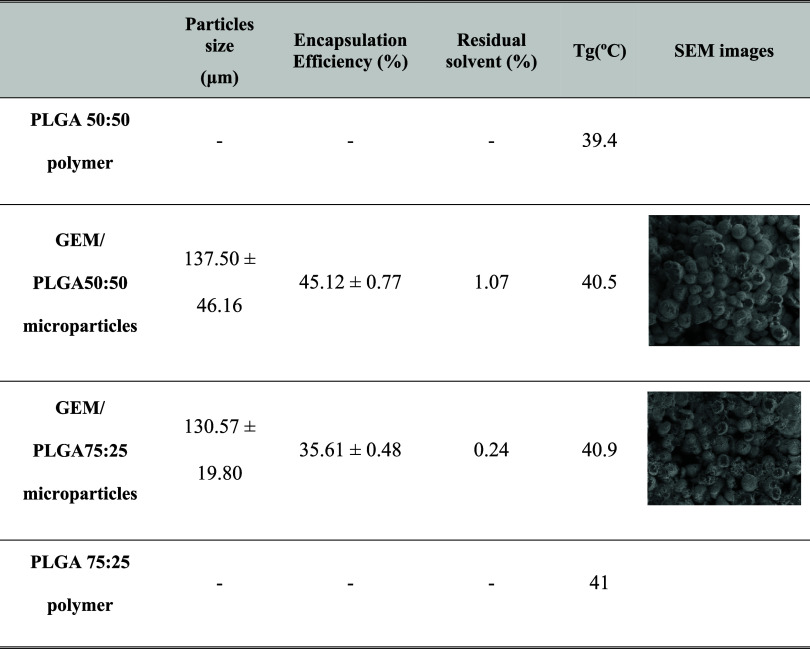
Microparticles Synthesized under Optimal Conditions^a^

a Solvent: EL; 1° step of agitation (rpm): 12,000; 2° step of agitation (rpm): 600 rpm; polymer concentration organic solution (% w/v): 1.5.

The glass transition temperature (*T*_g_) is one of the key physicochemical properties of the PLGA polymer. The *T*_g_ of PLGA typically lies above physiological temperature (37 °C) and increases with higher lactide content, molecular weight of the polymer, or the addition of compounds such as active pharmaceutical ingredients (APIs) or plasticizers.^45,46^ Since the average glass transition temperature (40 °C) of the microparticles (Table 5) was higher than the normal body temperature, their stability was deemed suitable for use as controlled release devices. This suggests that PLGA particles would not undergo a glass transition in a 37 °C drug release environment.

Last, the microparticles synthesized under optimal conditions exhibit an EL content of less than 1%. Moreover, it was found that this residual solvent can be completely removed if the microparticles undergo an additional freeze-drying step. It is important to note that EL is Generally Recognized As Safe (GRAS) and is commonly used as a solvent in the preparation of food additives and the pharmaceutical industry. Based on the optimized methodologies developed in this study, an in vitro release analysis will be essential to elucidate the underlying mechanisms governing drug release and polymer degradation and will be the focus of forthcoming research.

## 4 Conclusions

In this work, the production of PLGA microparticles was developed by using the solvent evaporation method with both traditional and green solvents. Two sets of experiments were carried out: one for the formation of nonloaded microparticles and another for producing microparticles encapsulating GEM. In the first case, the oil-in-water solvent evaporation method was employed, while for GEM encapsulation, a water-in-oil-in-water method was developed due to the hydrophilic nature of the drug.

GEM-loaded PLGA microparticles were successfully produced using the solvent evaporation method, applying both green solvents, ethyl lactate and ethyl acetate. The smallest and most homogeneous particles with the highest encapsulation efficiency were obtained with EL at higher polymer concentration (1.5% w/v) and 1200 rpm agitation, reaching 130.57 ±  19.80 μm with 35.61 ± 0.48% encapsulation efficiency. Although encapsulation efficiencies with EA were higher, above 50%, because of the higher particle size, the resulting ones were unsuitable for injectable formulations, and aggregation was more frequently observed compared with EL-based systems.

Overall, ethyl lactate proved to be the more effective solvent for producing GEM-loaded microparticles with smaller sizes and better uniformity despite slightly lower encapsulation efficiency values. The use of a homogenizer at 12,000 rpm allowed further refinement of particle size distribution. These results support the suitability of EL as a green solvent alternative for developing drug delivery systems with improved particle control and encapsulation quality.

Encapsulation efficiency was higher than that in previous studies due to the smaller size of the microparticles. Additionally, FTIR analysis confirmed the presence of GEM in the loaded microparticles, validating the success of the encapsulation process.
